# The Impact of Obesity on the Left Atrium and Arrhythmia Recurrence in Patients with Atrial Fibrillation Undergoing Ablation

**DOI:** 10.3390/jcm14197043

**Published:** 2025-10-05

**Authors:** Beata Uziębło-Życzkowska, Marek Kiliszek, Krystian Krzyżanowski, Paweł Krzesiński

**Affiliations:** Department of Cardiology and Internal Diseases, Military Institute of Medicine-National Research Institute, 04-141 Warsaw, Poland; mkiliszek@wim.mil.pl (M.K.); kkrzyzanowski@wim.mil.pl (K.K.); pkrzesinski@wim.mil.pl (P.K.)

**Keywords:** obesity, left atrial size, left atrial function, atrial fibrillation recurrence

## Abstract

**Objectives**: Obesity and atrial fibrillation (AF) are strongly linked and are both associated with significant left atrial (LA) pathology. This study aimed to assess differences in LA size and function between obese and non-obese AF patients and to evaluate AF recurrence in both groups. **Materials and Methods**: We retrospectively analyzed patients undergoing first-time ablation for AF. Obesity was defined as body mass index ≥30 kg/m^2^, and patients were divided accordingly into obese and non-obese groups. **Results**: Among 672 patients (median age of 66 years; 39.1% women), 308 (45.8%) were obese. Obese patients had significantly larger LA dimensions (LA area, LA volume, and LAVI indexed to height^2^ (but not that indexed to body surface area (BSA)); *p* < 0.001), as well as higher LA-pressure-related parameters (LA stiffness index (*p* = 0.004), E-wave velocity (*p* = 0.002), and E/e′ ratio (*p* < 0.001)) and invasively measured mean LA pressure (*p* < 0.0001). However, there were no significant differences in parameters directly reflecting LA function, such as LA emptying fraction, LA reservoir strain, or LA appendage velocity. These findings remained consistent in the sinus rhythm subgroup (n = 374). The 1-year AF recurrence rate did not differ between obese and non-obese groups (data available for 73.8% (496) patients; *p* = 0.40), regardless of baseline rhythm. **Conclusions**: In AF patients undergoing their first ablation, obesity was associated with a larger LA size and higher LA pressure. In obese individuals, indexing LA dimensions to height^2^ seems to better reflect LA enlargement than indexing to BSA. LA function and AF recurrence rates after a 1-year follow-up period were similar between obese and non-obese patients.

## 1. Introduction

Recently published meta-analyses have shown that each increase in body mass index (BMI) is associated with a significant rise in the risk of atrial fibrillation (AF) [1,2]. Obesity is strongly associated with left atrial (LA) enlargement, an important precursor of AF [3]. It is believed that LA enlargement in obese individuals occurs as a consequence of elevated arterial, left ventricular (LV), and LA filling pressures, leading to the development of diastolic dysfunction [4]. To date, several mechanisms contributing to obesity-mediated AF have been identified, including cardiac adiposity, inflammation, fibrosis, oxidative stress, ion channel remodeling, and autonomic dysfunction [5]. Regardless of the presence of obesity, LA dilatation is also the most common echocardiographic feature in patients with AF. As described by Leventopoulos et al. [6], structural remodeling of the LA involves atrial fibrosis, which leads to both structural and electrical changes that prolong conduction and promote reentry and rotor formation.

In addition to echocardiographic and invasive measures, cardiac magnetic resonance (CMR) offers valuable complementary insights, particularly in characterizing atrial fibrosis and adiposity, both of which may influence arrhythmic burden and ablation outcomes. Peer-Zada et al. [7] found that higher volumes of epicardial and pericardial adipose tissue measured using CMR are associated with impaired cardiac function in patients with paroxysmal AF. Similarly, Skoda et al. [8] demonstrated spatial overlap between areas of epicardial adipose tissue and atrial wall fibrosis on MRI, reinforcing the concept that localized adiposity may contribute directly to structural remodeling.

It, therefore, seems natural that the combination of obesity and AF would lead to an even more significant enlargement of the LA. At the same time, fibrosis in the LA wall, which occurs in both of the mentioned conditions [3,9], may lead to impaired LA function and, consequently, increased pressure within. The data on this topic, however, remain inconclusive.

Further research is also needed to explore the impact of obesity on AF treatment outcomes. It is reported that obesity can affect outcomes after ablation, such as increased AF recurrence [10]. However, the impact of obesity on catheter ablation, including outcomes, AF recurrence, and complications, is still being extensively evaluated.

Consequently, the aim of this study was to assess the differences in LA size and function in patients with AF according to the presence or absence of obesity. Although LA enlargement in obese individuals with AF is well established, data regarding its function in these conditions have not been precisely examined. An additional aim of our study was to assess the arrhythmia recurrence rate in the context of obesity.

## 2. Materials and Methods

### 2.1. Study Design and Patient Selection

Our study retrospectively analyzed collected data from all consecutive patients with AF who underwent first-time catheter ablation at a single cardiology center between March 2019 and January 2024. The only exclusions were suboptimal echocardiographic images, heart rate ≥ 120 bpm due to unreliable measurements of advanced echocardiographic parameters, mitral stenosis, and hypertrophic cardiomyopathy. All patients underwent echocardiographic examinations on the day of ablation. The heart rhythm (AF or sinus rhythm) was consistent during both the echocardiographic examination and the ablation procedure.

Additionally, demographic data, the results of basic laboratory tests, medical history of comorbidities, smoking status, and duration of arrhythmia were collected for all patients.

### 2.2. Echocardiographic Assessment

Transthoracic (TTE) and transoesophageal (TEE) echocardiographic examinations were performed immediately before catheter ablation using a high-quality echocardiography system (Vivid E95, General Electric, Horten, Norway). Measurements were made off-line using the EchoPac 204 workstation from recorded digital loops, ensuring a frame rate of 60–80 frames per second, especially when obtaining strain parameters. Standard echocardiographic measurements were performed, including linear and volumetric assessments of the LV, right ventricle, and LA, in accordance with current guidelines [11,12,13]. To estimate LAVI, two methods were used—indexing to body surface area (BSA), as recommended by current guidelines, and the proposed indexing to height squared (h^2^) [11,12,14]. To assess LA function, the following parameters were used: LA strain measurements, including reservoir strain (LASr) (in all patients), and additionally conduit (LAScd) and contraction strain (LASct) only in patients examined in SR; LA emptying fraction (LAEF), calculated using dedicated automated software for LA assessment (AFI LA); and left atrial appendage velocity (LAAV), evaluated in TEE. Given the irregular rhythm in AF, echocardiographic measurements were obtained from three consecutive cardiac cycles and subsequently averaged.

For all strain measurements, although some segments were excluded due to the inability to achieve adequate tracking, global strain values were calculated as the average of the remaining segments. The contraction strain index (CSI) was calculated using the formula CSI = (LASr/LASct) × 100. The onset of the QRS wave was used as the reference point for assessing LA strain values. Left atrial strain analysis was performed using automated software specifically designed for LA assessment (automated functional imaging of the left atrium (AFI LA). The left atrial emptying fraction (LAEF) was automatically calculated using AFI LA. The left atrial stiffness index (LASI) was assessed noninvasively and defined as the E/e′ ratio divided by LASr. The LAA emptying velocity was measured 1 cm from the LAA ostium and averaged from three measurements taken at two different scanning angles.

### 2.3. Left Atrial Pressure Measurements

The left atrium (LA) was accessed using a double transseptal puncture technique with an 8.5F transseptal sheath, a Swartz™ SL0, and a BRK™ needle (Abbott Medical, Plymouth, MN, USA). Left atrial pressure (LAP) was directly measured immediately after the transseptal puncture through the transseptal sheath, which was equipped with a specialized converter for invasive blood pressure measurement (B. Braun Melsungen AG, Melsungen, Germany). The system was integrated with the Philips Azurion image-guided therapy platform (Philips Medical Systems DMC GmbH, Hannover, Germany). The LAP values (max, min, and mean) were recorded automatically.

### 2.4. Ethical Statement

This study was conducted in accordance with good clinical practice guidelines and the Declaration of Helsinki. The study protocol was approved by the local ethics committee (Resolution No.18/24), which waived the requirement for obtaining informed consent due to the retrospective nature of the study.

### 2.5. Statistical Methods

The normality and distribution of continuous variables were evaluated using the Shapiro–Wilk test. These variables are presented either as means with standard deviations (SDs) or as medians with interquartile ranges (IQRs: 1st–3rd quartile), depending on their distribution. Categorical variables are reported as absolute counts and percentages. To compare two groups of continuous and categorical variables, independent *t*-tests, Mann–Whitney U tests, and chi-square tests were used. A two-tailed *p*-value of <0.05 was considered statistically significant. To compare groups in terms of the co-occurrence of independent variables, non-parametric tests (Mann–Whitney U) were applied, along with Spearman’s rank correlations for continuous variables. To better assess the strength of the observed differences, effect sizes (η^2^) were additionally calculated, allowing for a comparison of the relative influence of individual factors on the analyzed dependent variables. All analyses were performed using Stata Now 18.

## 3. Results

### 3.1. Study Population

**Figure 1:**
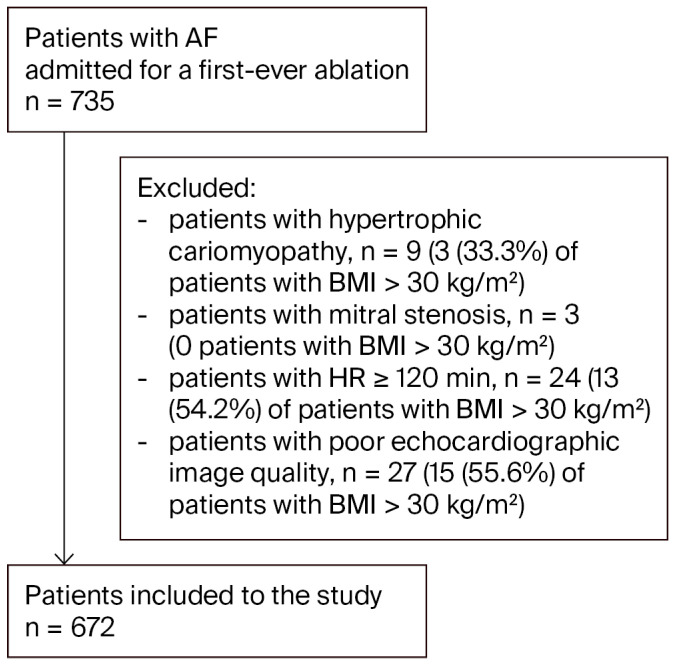
Study flow chart. Abbreviation: AF, atrial fibrillation.

Out of an initial population of 735 patients admitted for AF ablation, 672 were ultimately included in this study (Figure 1).

The median age of the included patients was 66 years (IQR 58–72), and the majority were male (61%). Obese patients made up 45.8% of the study group, with a mean BMI of 33.2 (IQR 31.3–36). BMI for the whole study group was 29.4 kg/m^2^ (IQR 26.4–32.8). The mean duration of AF before the first-ever catheter ablation was 3 years (IQR 1–5) for the whole study group, and 55.2% of patients presented with paroxysmal AF. The number of patients examined during AF was comparable between the two groups (with and without obesity) (*p* = 0.25), enabling a combined analysis of both groups.

The group of obese patients did not differ from the non-obese group in terms of age distribution, gender, arrhythmia duration, or heart rate during the echocardiographic examination, as detailed in Table 1. As expected, however, the obese group had a higher prevalence of comorbidities such as hypertension, diabetes, and heart failure (HF) with preserved ejection fraction (HFpEF). Table 1 provides a detailed comparison, including laboratory data for both groups.

### 3.2. Data from the Assessment of LA, LV, and RV Sizes

**Figure 2:**
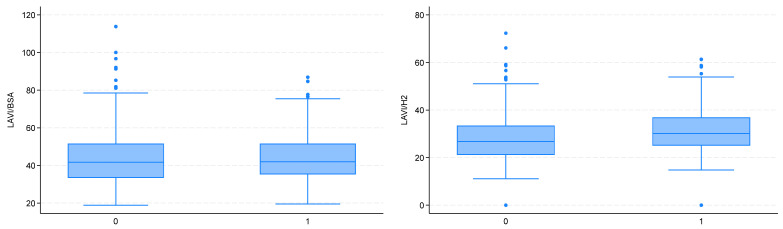
Left atrial enlargement estimated using the LAVI/BSA index (**left**) and the LAVI/H^2^ index (**right**) (0—non-obese patients; 1—obese patients).

Echocardiographic assessment revealed significantly larger LA dimensions in the obese group in terms of both linear measurements (LAd) and left atrial area (LAa). Particularly interesting and important findings were obtained in the evaluation of LA volume. As expected, the non-indexed LA volume (LAV) was significantly larger in the obese group. However, when indexed to BSA (as currently recommended), the LAV was comparable between the two groups. At the same time, LAV indexed to height squared (h^2^) was significantly higher in the obese group (Figure 2).

Similarly, the obese group exhibited significantly larger dimensions of the right ventricle, LV, and right atrium. Detailed echocardiographic and electrophysiological data are presented in Table 2.

### 3.3. Data from the Assessment of LA Function

The parameters reflecting LA function did not differ between the two groups. These included LA strain parameters, LAEF, and LAAV as assessed in TEE (Table 2).

### 3.4. Data from the Invasive and Noninvasive Assessment of LA Pressure

The LA pressure (LAP) values (maximum, mean, and minimum), assessed invasively and directly during the ablation procedure, were significantly higher in the obese group. Similarly, echocardiographic parameters that estimate LA pressure noninvasively also indicated elevated values, with significantly higher E/e′ avg ratio and LASI values observed in the obese group.

### 3.5. Subgroup Analysis

The analysis of data from the subgroup of patients in SR during the echocardiographic examination also showed no differences between obese and non-obese patients in terms of other LA systolic function parameters, such as LAScd, LASct, mitral inflow A-wave velocity, and myocardial a′ velocities from the medial and lateral mitral annulus.

Detailed results of the subgroup analysis of patients in SR are presented in Table S1.

### 3.6. Analysis of Co-Occurrence of Independent Variables

The interdependence analysis revealed the presence of confounding variables in the relationship between BMI and LA size. It was demonstrated that factors such as sex, hypertension, heart failure, and diabetes may both correlate with BMI and independently influence atrial structure. Notably, sex and heart failure showed a greater effect (η^2^ of 0.07–0.08 and 0.13–0.16, respectively) than BMI itself. Detailed results of this analysis are presented in Table 3.

### 3.7. AF Recurrence Rate in Study Population

**Figure 3:**
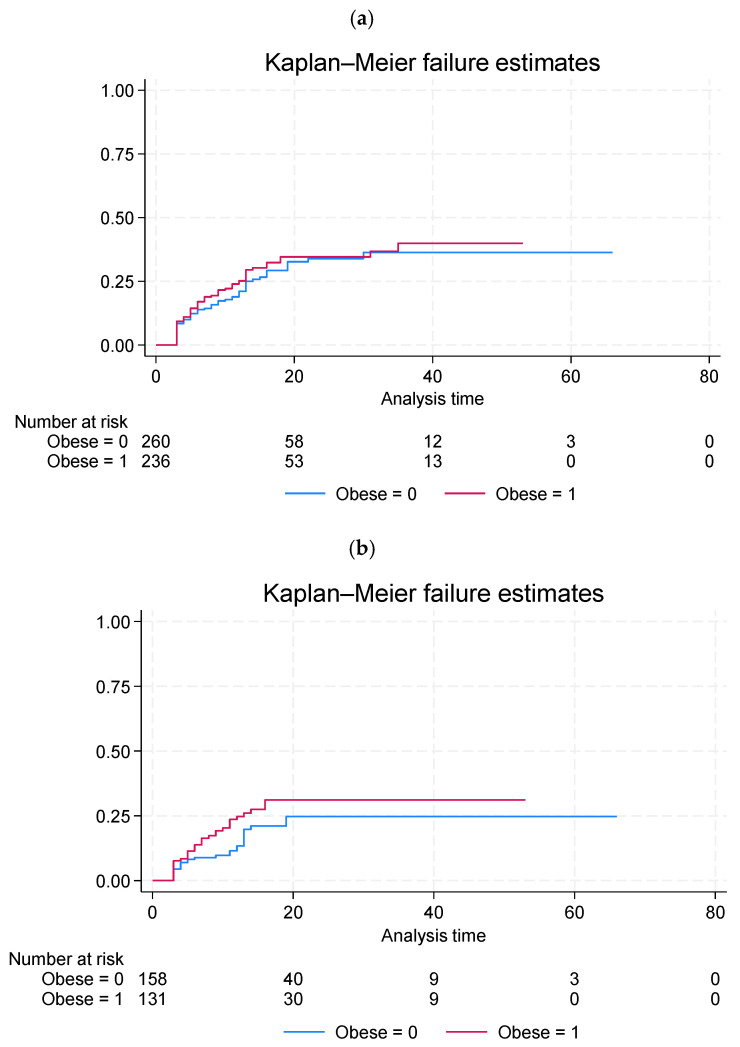
Kaplan–Meier failure curves showing the cumulative incidence of AF recurrence stratified by obesity status (**a**) in the entire study population and (**b**) in patients with sinus rhythm at baseline. Differences between groups were assessed using the log-rank test.

The analysis did not reveal any differences in the AF recurrence rate between the obese and non-obese groups after a mean follow-up period of 15 (±12) months. For more details, see Figure 3.

## 4. Discussion

The main findings of our study can be summarized as follows. Firstly, obese patients with AF undergoing their first-ever ablation exhibit enlarged LA dimensions and higher LA pressures, as assessed both invasively and noninvasively. It should also be noted that the results of our analysis suggest that LA size in obese patients should be indexed to height^2^ rather than to BSA. Secondly, LA function, evaluated using specific echocardiographic parameters, remains unimpaired. Finally, the AF recurrence rate does not differ between obese and non-obese patients.

The enlargement of the LA is a typical echocardiographic feature observed in both patients with AF [15] and obese patients [16]. The enlarged LA dimensions, already characteristic of AF patients, were found to be even more pronounced in the group of patients with obesity in our study. However, an issue regarding the estimation of LA size in obese patients concerns the method used to index its size. Current guidelines recommend indexing LA volume to BSA [12,14,17]. However, numerous studies confirm that this indexing method is inappropriate for obesity, as it underestimates LA structural remodeling in obese patients [13,18]. Aga et al. [14] demonstrated that significantly more patients with LA dysfunction (assessed by LA strain parameters) would be correctly identified using LA/height^2^ compared to LA/BSA (41.5% vs. 15.0%, *p* < 0.001). Many authors have proposed new methods for indexing LA in obese individuals, such as indexing to height^2^ [13] or the so-called ideal weight (based on height and sex) [19]. Our study confirms that obesity likely has a strong impact on LA classification, though this has not been thoroughly studied before. In our study, the group of obese patients with AF had a significantly enlarged LA assessed through echocardiographic parameters such as LAd, LA area, and LAV (*p* = 0.003 for LAd; *p* < 0.0001 for LA area and LAV), but not LAVI indexed to BSA (*p* = 0.37). In contrast, indexing LAV to height^2^ provided consistent results, demonstrating significantly higher LAVI values in obese patients (*p* < 0.0001). The study by Mulloy et al. [19] demonstrated that the change resulting from the new measurement of the LA volume index led to a reclassification of the degree of LA enlargement in many patients, nearly all of whom were reclassified into the group with more advanced LA enlargement. Our findings also support recent evidence suggesting that indexing LA volume to height^2^ may be more appropriate than indexing it to BSA in obese individuals. This has been demonstrated, for instance, in the ESC/EACVI case discussion [20], which showed that the choice of indexing method substantially changes the classification of LA enlargement in obese patients. Studies such as that by Salvetti et al. [21] demonstrated that height^2^ indexing identifies a higher prevalence of LA enlargement compared with BSA indexing, particularly in overweight and obese patients. Consistently, the 2024 ESC hypertension guidelines list LA volume indexed to height^2^ as a marker of hypertension-mediated organ damage, with specific sex-based thresholds (>18.5 mL/m^2^ for men and >16.5 mL/m^2^ for women) [22]. These observations raise the possibility that future echocardiographic and hypertension guidelines may increasingly adopt height^2^ indexing to reduce the risk of misclassification in this population.

Another significant finding of our study was the demonstration of elevated LAP values in the obese group, measured both directly during ablation and estimated noninvasively. In our study, echocardiographic parameters used for noninvasive estimation of LAP (E/e′ avg ratio and LASI) were significantly elevated in the obese group. LASI is a relatively new echocardiographic parameter that can be assessed noninvasively using the E/e′ ratio in conjunction with LASr [23]. A high concordance of LASI measurements with invasively estimated LA stiffness has been demonstrated [24]. This echocardiographic parameter has been associated with LA enlargement, LV diastolic dysfunction, and collagen synthesis [23]. Interestingly, it has been shown that in physiological LA enlargement, as observed in athletes, LASI values remain normal, indicating the potential use of this parameter in differentiating between physiological and pathological LA remodeling [25]. Previous studies have demonstrated that obesity contributes to increased arterial, LV, and LA filling pressures, ultimately promoting diastolic dysfunction [4,5]. Echocardiographic parameters such as E/e′ and LASI enable the noninvasive estimation of elevated LAP with high accuracy.

A rather surprising finding of our study was the absence of any differences in parameters assessing LA function. No differences were observed between obese and non-obese patients in terms of LASr (for the whole group), or in terms of LASr, LASct, LAScd, and CSI in the subgroup with sinus rhythm. Moreover, other parameters, such as LAEF and LAAV, also did not indicate impaired LA function in the obese group. In a recent study by Çöteli et al. [26], CSI was shown to correlate with atrial fibrosis assessed by CMR T1 mapping and to decrease during follow-up after AF ablation, suggesting its potential role as a marker of atrial remodeling. However, in our cohort, CSI assessed in patients in sinus rhythm did not differ between individuals with and without obesity (*p* = 0.21). This may reflect the limited sensitivity of CSI to obesity-related remodeling, but it may also be related to the relatively small sample size and warrants confirmation in larger studies. The study by Aga et al. [13] revealed significantly reduced LA function assessed by LASr, LAScd, and LASct in obese patients without known cardiovascular disease compared to non-obese individuals. Interestingly, the authors did not demonstrate any differences in the parameters of LV diastolic function. The authors conclude that, consequently, LA dysfunction revealed through LA strain measurements may serve as a predictor of HFpEF development. In our analysis, all patients already had reduced LAS values (compared to normal [27]) due to coexisting AF (see Table 2 and Table 3). This could explain the fact that AF, as a significant pathology of the LA, reduces LA strain parameters to such an extent that the impact of obesity is no longer evident. Our previous studies showed that LAS values in AF patients are significantly reduced compared to normal values [28,29]. The reason for obtaining such a result remains unclear, especially considering that in our previous study, we demonstrated that low LASr and LASct values, as well as a high E/e′ ratio, were associated with elevated LAP in AF patients [28]. Our findings regarding the absence of significant differences in LA function between obese and non-obese patients should be interpreted with caution. In AF, the sensitivity of atrial strain parameters is limited, and in our cohort, full strain phase analysis (including LAScd and LASct) was feasible only in patients in sinus rhythm. This methodological limitation may have influenced our results. Furthermore, the concept of atrial cardiomyopathy has recently been emphasized as a unifying framework integrating structural, electrical, and functional alterations of the atria, which predispose to AF and thromboembolic complications [30]. Importantly, obesity has been identified as a major contributor to atrial cardiomyopathy through hemodynamic overload, metabolic dysregulation, inflammation, and atrial fibrosis driven by epicardial adipose tissue. For this reason, the absence of significant differences in LA functional parameters between obese and non-obese patients needs to be considered carefully. While it is possible that AF itself exerts such a strong adverse effect on atrial strain that the impact of obesity is masked, this explanation remains speculative. Our findings are therefore hypothesis-generating and require validation in larger studies with appropriate rhythm stratification.

Although patients with obesity exhibited significantly larger LA area and volume, the presence of potential confounding variables must be considered. Factors such as sex, hypertension, HF, and diabetes may correlate with BMI and independently affect LA structure. In particular, sex and HF showed a greater effect (η^2^ of 0.07–0.08 and 0.12–0.16, respectively) than BMI alone. Therefore, the observed association between obesity and LA structure should be interpreted with caution, as it may be partially dependent on coexisting clinical conditions. It is important to note, however, that the sex distribution did not differ significantly between the obese and non-obese groups (*p* = 0.86). In contrast, HF was significantly more prevalent among obese patients (*p* = 0.0008), but this difference was limited to HFpEF (*p* = 0.03). As obesity is known to be closely linked with the occurrence of HFpEF, these two conditions cannot be effectively separated or analyzed independently. Other echocardiographic parameters related to LA pressure—such as the E wave, E/e′ average, and mean LAP—were not significantly influenced by confounding variables. Taken together, these findings indicate that the association between obesity and LA remodeling should not be interpreted as causal, since comorbidities such as HF, hypertension, and diabetes—conditions closely linked to obesity—likely contribute substantially to the observed changes. This confounding effect underscores the complexity of separating the independent role of obesity in atrial remodeling.

Finally, in the current study, we did not identify any differences in AF recurrence between obese and non-obese patients. These study results are based on a relatively small number of patients, as data on arrhythmia recurrence were obtained only for a limited subset of the cohort (73.8%–496 patients) and warrants careful consideration. An earlier large study investigating the impact of BMI on ablation outcomes showed that patients with baseline BMI ≥ 30 kg/m^2^ experienced higher rates of AF recurrence at 12-month follow-up, while procedural duration and radiation exposure were increased in overweight and obese individuals [31]. A recently published large study involving 5841 patients demonstrated that although the efficacy of ablation decreased with increasing BMI, this was significant only for the group with Class III obesity (BMI ≥ 40 kg/m^2^) [32]. Class III obesity was independently associated with an increased risk of AF recurrence (hazard ratio: 1.30; confidence interval: 1.06–1.60; *p* = 0.01), while other groups had a similar risk to patients with normal weight. In conclusion, the authors emphasized that due to the low complication rate across all BMI groups, ablation should still be considered for individuals with morbid obesity (BMI > 40), as it improves quality of life. However, wherever possible, a formal weight management program and potentially bariatric surgery should be implemented prior to the procedure. Beyond lifestyle and procedural approaches, pharmacological strategies may also play a role in modulating AF risk in obese patients. Recent evidence suggests that sodium–glucose cotransporter-2 (SGLT2) inhibitors, widely used in the management of diabetes and heart failure, may reduce the risk of AF recurrence [33]. This highlights the potential of metabolic therapies as adjunctive measures to address atrial remodeling in obesity.

## 5. Study Limitations

This study has several limitations that should be acknowledged. First, it was conducted at a single center, which may limit the generalizability of the findings to broader populations. Second, the retrospective nature of the analysis may introduce selection bias and limits the ability to establish causal relationships. Despite these limitations, a major strength of the study is the relatively large sample size, which increases the statistical power of the analysis and supports the robustness of the observed associations. A potential selection bias cannot be fully excluded, as 27 patients, 55.6% of whom had obesity (BMI > 30 kg/m^2^), were excluded due to suboptimal echocardiographic image quality. However, this group represents only 3.7% of the initial cohort and 8.6% of the overall exclusions, limiting the extent of its influence on the study outcomes. One key limitation of this analysis was the inability to apply multivariate regression due to violations of key assumptions. Attempts to fit multivariable-adjusted models revealed non-random patterns and heteroscedasticity in residual diagnostics, with fitted-versus-residual plots indicating systematic, non-random errors. The Shapiro–Wilk test additionally confirmed significant deviations from normality of residuals. Additional checks further identified outlying observations (maximum Mahalanobis distance of 22.4) and considerable collinearity between several clinical covariates, such as the associations between heart failure and arrhythmia, or between age and hypertension. These issues undermined the stability and interpretability of multivariable models and precluded reliable estimation of independent effects. For this reason, non-parametric tests (Mann–Whitney U) and Spearman’s correlations were selected as more robust alternatives, with effect sizes (η^2^) calculated to estimate the strength of observed associations. Nevertheless, the absence of multivariable regression means that residual confounding cannot be fully excluded. In particular, we cannot rule out that factors such as age, sex, or comorbidities may have contributed to the observed relationships. Accordingly, our findings should be interpreted as descriptive associations rather than as definitive evidence of independent effects. Importantly, the statistical approach and its limitations were reviewed and confirmed by an independent biostatistics expert. Finally, the findings on AF recurrence in obese versus non-obese patients are based on a reduced sample size, as data on arrhythmia recurrence were available only for a subset of the cohort (73.8%; 496 patients), and should therefore be interpreted with caution.

## 6. Conclusions

In patients with AF undergoing first-time ablation, obesity was linked to larger LA size and higher pressure, without impairing LA function or affecting AF recurrence. Indexing LA size to height^2^ better reflected LA enlargement in obese individuals. The associations between obesity and LA structure may be partly influenced by comorbidities such as heart failure (particularly HFpEF), hypertension, diabetes, and age. Notably, HF had a stronger effect on LA remodeling than BMI alone. These findings emphasize the importance of considering clinical context when evaluating the impact of obesity on LA structure and support the use of alternative indexing methods in obese patients.

## Figures and Tables

**Table 1:** The basic characteristics of the study group—demographic, laboratory, and comorbidity data.

	All Group (n = 672)		
	Obese BMI ≥ 30 kg/m^2^ n = 308 (45.8%)	Non-Obese BMI < 30 kg/m^2^ n = 364	*p*
BMI, median (IQR)	33.2 (31.3–36)	26.8 (24.7–28)	
Demographic data			
Age, years, median (IQR)	65 (57.5–70.5)	67 (58.5–73)	0.08
Female gender, n (%)	119 (38.6)	143 (39.3)	0.86
HR (/min)	70 (62–86)	70 (62–87)	0.78
AF during echocardiography	104 (33.8)	151 (41.5)	0.25
Time from first AF diagnosis, years, median (IQR)	3 (1–5)	3 (1–6)	0.91
Laboratory data			
Hemoglobin, g/dL	14.7 (13.6–15.6)	14.2 (13.3–15.2)	0.002
WBC, tys/uL	7.2 (6–8.7)	6.8 (5.7–8.1)	0.001
Creatinine, mg/dL	1 (0.9–1.2)	1 (0.8–1.1)	0.03
eGFR, mL/min/m^3^	90 (59.5–90)	90 (61–90)	0.55
Clinical data, n(%)			
Hypertension	268 (87)	241 (66.2)	<0.0001
Diabetes mellitus	80 (26)	65 (17.9)	0.01
Stroke	16 (5.2)	21 (5.8)	0.87
Coronary artery disease	85 (27.6)	86 (23.6)	0.28
Smoking	80 (26)	88 (24.2)	0.61
HF	117 (38)	93 (25.5)	0.0008
HFrEF	32 (27.4)	31 (33.3)	0.49
HFmrEF	33 (28.2)	27 (29)	0.17
HFpEF	51 (43.6)	38 (40.9)	0.03

Abbreviations: AF, atrial fibrillation; BMI, body mass index; eGFR, estimated glomerular filtration rate; HF, heart failure; HFrEF, heart failure with reduced ejection fraction; HFmrEF, heart failure with mildly reduced ejection fraction; HFpEF, heart failure with preserved ejection fraction; HR, heart rate; WBC, white blood cells.

**Table 2:** Detailed echocardiographic and electrophysiological data of the two groups in the study population.

	Whole Group (n = 672)		
	Obese BMI ≥ 30 kg/m^2^ n = 308 (45.8%)	Non-Obese BMI < 30 kg/m^2^ n = 364	*p*
LVDd, mm	51 (48–55.5)	49 (46–53)	<0.0001
LVSd, mm	35 (32–40)	33 (30–37)	<0.0001
RVDd, mm	32 (30–33.6)	30 (28–32)	<0.0001
LVMI, g/m^2^	126 (104.5–152.3)	111.3 (96.3–133.7)	<0.0001
E, cm/s	74.5 (62.5–88)	70 (57.5–82)	0.002
e’ medial, cm/s	7 (6–8)	7 (6–9)	0.05
e’ lateral, cm/s	9 (7–11)	9 (8–12)	0.09
e’ average, cm/s	8.5 (6.5–9.5)	8.5 (7–10)	0.09
E/e′ average	8.94 (7.3–11.4)	8.3 (6.4–10.5)	<0.001
LV EF, %	60 (50–63)	60 (53–65)	0.01
LAd, mm	46.4 (43.4–50.5)	43.7 (40.7–46.9)	0.0025
LA area, cm^2^	27.3 (24–31.5)	25.2 (21.3–29.2)	<0.0001
RA area, cm^2^	19.6 (16.3–23.4)	18 (15–21.1)	<0.0001
LAV, mL	89 (75–110)	79.3 (63.2–101)	<0.0001
LAVI [BSA], mL/m^2^	41.9 (35.2–51.5)	41.7 (33.5–51.6)	0.37
LAVI [h^2^], mL/m^2^	30.2 (25–36.9)	26.8 (21.2–33.4)	<0.0001
LAEF, %	37 (25–50)	38 (24–50)	0.81
LAAV, cm/s	42 (30–60)	45.5 (29.5–71)	0.09
TRV, m/s	2.4 (2.2–2.7)	2.4 (2.1–2.6)	0.07
SEC, n (%)	39 (12.7)	36 (9.9)	0.29
Speckle tracking echocardiography data—left atrial and left ventricular function parameters			
LASr, %, median (IQR)	14 (9–23)	16 (10–24)	0.2
LV GLS, %, median (IQR)	17.3 (13.8–20.2)	17.8 (14.1–20.5)	0.2
LASI (E/e′/LASr), median (IQR)	0.63 (0.34–1.09)	0.51 (0.29–0.96)	0.004
Electrophysiological data, median (IQR)			
LAP max	23 (17–28)	19 (15–25)	<0.0001
LAP med	15 (12–19)	13 (10–17)	<0.0001
LAP min	10 (7–13)	9 (6–11)	<0.0001

Abbreviations: LA, left atrial; LAAV, left atrial appendage emptying velocity; LAd, left atrial antero-posterior dimension; LAEF, left atrial emptying fraction; LAP, left atrial pressure; LASI, left atrial stiffness index (E/e′/LASr); LASr, left atrial strain during reservoir phase; LAV, left atrial volume; LAVI, left atrial volume index; LVDd, left ventricular diastolic diameter; LVSd, left ventricular systolic diameter; LV EF, left ventricular ejection fraction; LV GLS, left ventricular global longitudinal strain; LVMI, left ventricular mass index; RA, right atrial; RVDd, right ventricular diastolic diameter; SEC, spontaneous echocardiographic contrast; TRV_,_ peak tricuspid regurgitation velocity.

**Table 3:** The results of the analysis of the co-occurrence of potential confounding variables (effect size interpretation (η^2^): <0.01—negligible/no effect; 0.01–0.05—small effect; 0.06–0.13—medium effect; >0.13—large effect).

Variables	LA Area (cm^2^)	LAV (mL)	LAVI/H^2^ (mL/m^2^)	LASI	E Wave (cm/s)	E/e′ Avg Ratio	LAP Mean
BMI							
*p*	<0.001	<0.001	<0.001	<0.001	<0.001	<0.001	<0.001
η^2^	0.07	0.08	0.09	0.06	0.05	0.06	0.05
Sex							
*p*	<0.001	<0.001	0.33	0.008	<0.001	<0.001	0.23
η^2^	0.07	0.08	0.001	0.01	0.03	0.07	0.002
HT							
*p*	<0.001	<0.001	<0.001	0.004	0.009	<0.001	<0.001
η^2^	0.02	0.02	0.03	0.01	0.01	0.02	0.02
DM							
*p*	0.009	0.005	<0.001	<0.001	<0.001	<0.001	<0.001
η^2^	0.01	0.01	0.02	0.04	0.02	0.06	0.04
CKD							
*p*	0.28	0.22	0.003	0.002	0.06	0.001	0.66
η^2^	0.002	0.002	0.01	0.02	0.005	0.02	0
CAD							
*p*	0.001	0.001	<0.001	<0.001	0.17	<0.001	0.33
η^2^	0.02	0.02	0.02	0.04	0.003	0.04	0.001
HF							
*p*	<0.001	<0.001	<0.001	<0.001	0.006	<0.001	<0.001
η^2^	0.13	0.14	0.12	0.16	0.01	0.07	0.04

Abbreviations: BMI, body mass index; CAD, coronary artery disease; CKD, chronic kidney disease; DM, diabetes mellitus; HF, heart failure; HT, hypertension; LA, left atrial; LAP, left atrial pressure; LASI, left atrial stiffness index (E/e′/LASr); LAV, left atrial volume; LAVI, left atrial volume index.

## Data Availability

The datasets are available from the corresponding author upon reasonable request.

## Supplementary Materials

The following supporting information can be downloaded at https://www.mdpi.com/article/10.3390/jcm14197043/s1, Table S1: Detailed data for both obese and non-obese patients with SR during procedures.
