# Supplementary material for: The Impact of Obesity on the Left Atrium and Arrhythmia Recurrence in Patients with Atrial Fibrillation Undergoing Ablation
Source: J Clin Med. 2025 Oct 5;14(19):7043. doi: 10.3390/jcm14197043 (PMC12524957; doi:10.3390/jcm14197043)
Supplement: Supplementary file 1 [file jcm-14-07043-s001.zip › jcm-3884694-supplementary.pdf]

	SR group (n=374)		
	Obese BMI≥30kg/m^2 n=161 (43%)	Non-obese BMI<30kg/m^2 n=213 (57%)	p
With AF recurrence	32(28.1) (n=114)	42 (29.2) (n=144)	0.45
BMI, median (IQR)	33 (31.2-35.7)	26.7 (24.5-28)	
**Demographic data**			
Time from first AF diagnosis, years, median (IQR)	3 (2-5)	2.5 (1-5)	0.34
Age, years, median (IQR)	65 (56-70)	65 (56-72)	0.36
Female gender, n (%)	73 (45.3)	94 (44.1)	0.83
**Echocardiographic data, median (IQR)**			
LVDd, mm	51 (48.6-54)	49.7 (47-53)	0.003
LVDs, mm	34 (32-26)	33 (30-36)	0.004
RVDd, mm	31.3 (30-33)	30 (28-32)	<0.0001
LVMI, g/m^2	119.1 (100.7-144.5)	108.6 (95.2-131.6)	0.002
E, cm/s	68 (56.5-81)	64 (53-78)	0.07
A, cm/s	66.5 (53-78)	62 (50-76)	0.11
E/A	1.05 (0.82-1.27)	1 (0.79-1.4)	0.86
e' medial, cm/s	6 (5-8)	7 (6-8)	0.02
e' lateral, cm/s	9 (7-10)	9 (8-11)	0.09
e' average, cm/s	7.5 (6.5-9)	8 (6.5-9.5)	0.04
E/e' average	8.7 (7.2-11.1)	7.9 (6.2-10)	0.003
a'm, cm/s	8 (6-9)	8 (7-10)	0.33
a'l, cm/a	9 (7-11)	9 (7-11)	0.7
LV EF, %	62 (59-65)	63 (58-66)	0.08
LAd	44.7 (40-47.7)	41.6 (38.1-44.6)	0.04
LA area, cm^2	25 (22.4-27.6)	23.1 (20.3-26.1)	<0.0001
RA area, cm^2	17 (15.4-19.8)	16.6 (14.5-19)	0.03
LAV, ml	79 (67-95)	71.5 (59.9-87)	<0.0009
LAVI, ml/m^2	36.9 (33-44.8)	37.5 (31.9-45)	0.81
LAVI [h^2], ml/m^2	25.8 (23.1-31.4)	23.6 (19.8-29.2)	<0.0001
LAEF, %	49 (42-58)	48 (40-55)	0.13
LAAV, cm/s	56 (41-75)	61 (42-80)	0.19
TRV, m/s	2.4 (2.1-2.7)	2.3 (2.1-2.6)	0.24
SEC, n (%)	7 (4.3)	6 (2.8)	0.41
PA-TDI, ms	159 (147-174)	156 (143-168)	0.07
**Speckle tracking echocardioraphy data - left atrial and left ventricular function parameters**			
LASr, %, median (IQR)	23 (18-28)	23 (17.5-28)	0.7
LAScd, %, median (IQR)	11 (8-14)	11 (8-13)	0.9
LASct, %, median (IQR)	12 (9-15)	12 (8-15)	0.59
CSI, (LASct/LASr x100), median (IQR)	52.4 (46.2-60)	50 (42.1-58.3)	0.21
LV GLS, %, median (IQR)	19.6 (17.6-20.9)	19.9 (17.7-21.6)	0.33
LASI (E/e'/LASr), median (IQR)	0.47 (0.37-0.68)	0.33 (0.24-0.52)	0.04
**Electrophysiological data, median (IQR)**			

LAP max	21 (16-26)	18 (15-22)	<0.001
LAP med	14 (11-17)	12 (10-15)	<0.001
LAP min	9 (6-12)	8 (5-10)	0.002
**Laboratory data, median (IQR)**			
Hemoglobin, g/dl	14.4 (13.3-15.1)	14.1 (13.1-15.1)	0.15
WBC, tys/ul	6.9 (5.7-8.3)	6.7 (5.7-7.9)	0.14
Creatinine, mg/dl	1 (0.8-1.1)	0.9 (0.8-1.1)	0.48
eGFR, ml/min/m^3	90 (78-90)	90 (65-90)	0.54
**Clinical data, n(%)**			
Hypertension	137 (85.1)	139 (65.3)	<0.0001
Diabetes mellitus	43 (26.7)	34 (16)	0.01
Stroke	6 (3.7)	11 (5.2)	0.62
Coronary artery disease	41 (25.5)	46 (21.6)	0.22
Smoking	42 (26.1)	50 (23.5)	0.39
HF	42 (26.1)	38 (17.8)	0.04
HFrEF	5 (11.9)	9 (23.7)	0.6
HFmrEF	10 (23.8)	9 (23.7)	0.48
HFpEF	27 (64.3)	20 (52.6)	0.04

Abbreviations: AF, atrial fibrillation; BMI, body mass index; eGFR, estimated glomerular filtration rate; HF, heart failure; HFrEF, heart failure with reduced ejection fraction; HFmrEF, heart failure with mildly reduced ejection fraction; HFpEF, heart failure with preserved ejection fraction; HR, heart rate; LA, left atrial; LAAV, left atrial appendage emptying velocity; LAd, left atrial antero-posterior dimension; LAEF, left atrial emptying fraction; LAP, left atrial pressure; LASI, left atrial stiffness index (E/e'/ LASr); LASr, left atrial strain during reservoir phase; LAV, left atrial volume; LAVI, left atrial volume index; LVDd, left ventricular diastolic diameter; LVSd, left ventricular systolic diameter; LV EF, left ventricular ejection fraction; LV GLS, left ventricular global longitudinal strain; LVMI, left ventricular mass index; RA, right atrial; RVDd, right ventricular diastolic diameter; SEC, spontaneous echocardiographic contrast; SR, sinus rhythm; TRV, peak tricuspid regurgitation velocity. WBC, white blood cells.

Table S1. Detailed data for both obese and non-obese patients with SR during procedures.
